# Fanconi syndrome induced by zoledronic acid: Two case reports

**DOI:** 10.1097/MD.0000000000048113

**Published:** 2026-03-27

**Authors:** Yanrong Chen, Jinhui Ma, Tao Ma, Yuqi Li, Lili Qiao, Yao Zhang

**Affiliations:** a Department of Urology and Office of Academic Research, Affiliated Hospital of Hebei University, Baoding, Hebei Province, China; b Department of Endocrinology and Office of Academic Research, Affiliated Hospital of Hebei University, Baoding, Hebei Province, China.

**Keywords:** zoledronic acid, bisphosphonate, fanconi syndrome, bone metastasis

## Abstract

**Rationale:**

Zoledronic acid (ZA) is a potent third-generation bisphosphonate widely used to manage bone metastases in prostate cancer. Although effective, ZA is associated with nephrotoxicity, which in rare cases may lead to Fanconi syndrome (FS): a form of acquired proximal renal tubular dysfunction. Clinicians may overlook early signs of FS, especially in patients receiving long-term bisphosphonate therapy.

**Patient concerns:**

We report 2 cases of prostate cancer complicated by FS following ZA administration. Patient #1, a 74-year-old male without prior renal dysfunction, developed hypouricaemia 4 months after initiating ZA, and was diagnosed with FS 2 years later. Patient #2, a 66-year-old male with chronic hepatitis B and long-term low-dose adefovir use, exhibited progressive tubular injury shortly after ZA initiation and was diagnosed with FS 4 years later.

**Diagnoses:**

Both patients were diagnosed with acquired FS based on laboratory abnormalities including hypophosphatemia, hypouricaemia, glycosuria, proteinuria, and metabolic acidosis. Differential diagnoses including hereditary FS and other nephrotoxic exposures were excluded.

**Interventions:**

In both patients, ZA was discontinued. Patient #1 was switched to denosumab, and received oral phosphate, potassium, and bicarbonate replacement. Patient #2 also discontinued adefovir and was transitioned to entecavir, alongside targeted supportive therapy with phosphate and vitamin D supplementation.

**Outcomes:**

Renal tubular function improved in both patients following drug withdrawal. Hypouricaemia, glycosuria, electrolyte abnormalities, and acid-base imbalance gradually resolved over 1 to 3 months, supporting a causal relationship between ZA and FS.

**Lessons:**

These cases highlight the importance of early recognition and close monitoring of renal tubular function in patients receiving ZA. Screening for FS, including urinalysis, blood gas analysis, and serum electrolytes, should be considered within the first month of therapy and repeated regularly thereafter. Clinicians should maintain high vigilance for FS, especially when ZA is used concurrently with other nephrotoxic agents such as adefovir.

## 1. Introduction

Prostate cancer is among the most prevalent malignant tumors in men.^[1]^ Bone metastasis is a frequent complication in patients with prostate cancer; it compromises bone integrity and often leads to skeletal-related events (SREs), including pathologic fractures, spinal cord compression, and intractable bone pain. SREs diminish patients’ quality of life while elevating their financial burden and mortality risk.^[2]^ Therefore, the prevention and management of bone metastases in prostate cancer are of paramount importance. Zoledronic acid (ZA) is widely administered for prostate cancer bone metastases and has demonstrated substantial efficacy in reducing or delaying SREs.^[3]^ In recent years, Fanconi syndrome (FS) has been increasingly reported following ZA administration in clinical settings.^[4,5]^ FS is a renal disorder characterized by proximal tubular dysfunction, resulting in excessive urinary loss of amino acids, glucose, bicarbonate, phosphate, uric acid, and electrolytes. Clinically, it presents with glycosuria, aminoaciduria, hyperchloremic metabolic acidosis, hypokalemia, hypouricemia, bone pain, skeletal deformities, fractures, and fatigue. If left untreated, it may lead to progressive skeletal abnormalities, declining renal function and chronic kidney disease.^[6]^ Here, we report 2 cases of prostate cancer in which patients developed FS following ZA treatment.

## 2. Case reports

Patient #1, a 74-year-old male, was diagnosed with prostate cancer and multiple bone metastases in May 2022 at Hebei University Hospital. He received 10.8 mg of goserelin acetate via monthly subcutaneous extended-release implant, 1000 mg of abiraterone acetate orally once daily, 5 mg of prednisone acetate orally once daily, and 4 mg of ZA intravenously once a month. In September 2022, he was found to have hypouricaemia, with a serum uric acid level of 175 µmol/L (reference range: 208–428 µmol/L). In October 2023, urinalysis revealed urinary protein (1+) and urinary glucose (1+). In March 2024, laboratory tests showed hypokalemia (serum potassium: 2.9 mmol/L; reference: 3.5–5.3 mmol/L), hyperchloremia (chloride: 113 mmol/L; reference: 99–110 mmol/L), hypophosphatemia (phosphate: 0.67 mmol/L; reference: 0.85–1.51 mmol/L), and markedly decreased serum uric acid ( < 89 µmol/L; reference: 208–428 µmol/L). Urinalysis indicated urinary protein (2+) and urinary glucose (3+). Arterial blood gas analysis showed a pH of 7.37 (reference: 7.35–7.45), with normal parathyroid hormone and alkaline phosphatase levels. He was diagnosed with FS. ZA was discontinued and replaced with denosumab for the management of bone metastases. Sodium bicarbonate, potassium citrate, calcium carbonate, and a phosphate mixture were administered to treat FS. 2 months after initiating treatment, his renal function improved, electrolyte levels normalized, urinary glucose became negative, and hypouricaemia showed marked improvement, as shown in Table 1. He remains under outpatient follow-up with stable renal function.

**Table 1:** Laboratory data for patient #1.

	At the start of ZA application	At the diagnosis of FS	After the discontinuation of ZA	Reference range
K (mmol/L)	3.9	2.9	4.2	3.5–5.3
Na (mmol/L)	140	142	141	137–147
Cl (mmol/L)	101	113	107	99–110
P (mmol/L)	1.28	0.67	0.98	0.85–1.51
Serum uric acid (µmol/L)	336	89	159	208–428
Creatinine (µmol/L)	59	109	96	58–110
Urine glucose	–	3+	Negative	Negative
Urine protein	–	2+	Negative	Negative

Cl = chloride, FS = fanconi syndrome, K = potassium, Na = sodium, P = phosphorus, ZA = zoledronic acid.

Patient #2, a 66-year-old male, had a history of chronic hepatitis B for over 20 years and had been taking oral adefovir dipivoxil 10 mg daily. In February 2018, laboratory tests revealed urinary protein (2+), hypophosphatemia (phosphate: 0.65 mmol/L; reference: 0.85–1.51 mmol/L), and hypouricaemia (uric acid: 127 µmol/L; reference: 208–428 µmol/L). In March 2018, following the diagnosis of prostate cancer at the Affiliated Hospital of Hebei University, he underwent radical prostatectomy. Postoperatively, he began oral bicalutamide 50 mg once daily and subcutaneous goserelin microspheres 3.6 mg once monthly. In January 2020, emission computed tomography of the thoracic spine, multiple ribs, and right hip joint revealed abnormally intense foci suggestive of altered bone metabolism, raising suspicion of bone metastasis. He was diagnosed with prostate cancer bone metastasis, and subsequently received monthly intravenous ZA injections (4 mg). He gradually developed bilateral lower limb weakness. In April 2020, urinalysis revealed urinary glucose (2+), while serum chloride was elevated at 113 mmol/L (reference: 99–110 mmol/L). He remained positive for urinary protein, hypophosphatemia, and hypouricaemia. In February 2023, he experienced marked sternal and sacral pain, accompanied by worsening fatigue. In July 2023, blood gas analysis showed a pH of 7.24 (reference: 7.35–7.45), and serum creatinine was slightly elevated at 115 µmol/L (reference: 57–111 µmol/L). In January 2024, laboratory tests revealed the following: potassium 3.4 mmol/L (3.5–5.3), chloride 115 mmol/L (99–110), calcium 2.12 mmol/L (2.11–2.52), phosphorus < 0.32 mmol/L (0.85–1.51), alkaline phosphatase 214 U/L (45–125), uric acid < 89 µmol/L (208–428), creatinine 106 µmol/L (57–111), urinary protein (2+), urinary glucose (3+), urinary pH 7.5, blood pH 7.25 (7.35–7.45), 24-hour urinary calcium 0.39 mmol/L (2.11–2.52), urinary chloride 114 mmol/24 hours (170–250), sodium 321 mmol/24 hours (130–260), potassium 54 mmol/24 hours (25–100), fractional excretion: phosphorus 31.12%, sodium 5.18%, potassium 34.3%, parathyroid hormone 351 pg/mL (9–101), bone-type alkaline phosphatase 41.98 ng/mL ( ≤ 20.1), 25-dihydroxy-vitamin D 22 ng/mL (30–100), osteocalcin 27.93 ng/mL (14–70), β-CTX 1.77 ng/mL (0–0.85), and NTX 160.90 ng/mL (15.13–76.31). Positron Emission Tomography/Computed Tomography revealed postoperative changes of prostate cancer without abnormal hypermetabolism in the operative region. Based on the above findings, he was diagnosed with FS. ZA and adefovir dipivoxil were discontinued, and entecavir was initiated for hepatitis B management. Oral phosphate solution, calcitriol, and sodium bicarbonate were prescribed for FS. One month after initiating treatment, the patient’s pain and fatigue showed marked improvement. After 3 months of treatment, his electrolyte levels normalized, hypouricaemia and acidosis significantly improved, and urinary glucose became negative (Table 2). He continues to receive outpatient treatment with stable clinical status.

**Table 2:** Laboratory data for patient #2.

	At the start of ZA application	At the diagnosis of FS	After the discontinuation of ZA	Reference range
K (mmol/L)	4.0	3.4	3.8	3.5–5.3
Na (mmol/L)	140	137	137	137–147
Cl (mmol/L)	109	115	109	99–110
P (mmol/L)	0.65	<0.32	1.02	0.85–1.51
Serum uric acid (µmol/L)	127	<89	91	208–428
Creatinine (µmol/L)	87	106	101	58–110
Urine glucose	–	3+	Negative	Negative
Urine protein	2+	2+	2+	Negative
Blood pH	–	7.25	7.34	7.35–7.45

Cl = chloride, FS = fanconi syndrome, K = potassium, Na = sodium, P = phosphorus, ZA = zoledronic acid.

## 3. Discussion

Acquired FS has been linked to exposure to certain drugs and heavy metals, with cisplatin, ifosfamide, tenofovir, and adefovir being among the most commonly implicated agents.^[6]^ Several cases of adefovir-induced FS have been reported,^[7,8]^ as well as case reports implicating ZA in FS.^[4,9,10]^ However, there are no known reports linking goserelin, abiraterone acetate, prednisone acetate, or bicalutamide to FS. Patient #1 developed hypouricaemia 4 months after initiating ZA therapy. With continued use, he exhibited significant renal tubular dysfunction, evidenced by hypophosphatemia, persistent hypouricaemia, hyperchloremia, proteinuria, and glycosuria: all of which strongly correlated with ZA exposure. Following ZA discontinuation, his electrolyte levels normalized, urinary glucose turned negative, and hypouricaemia markedly improved. Patient #2 had been on long-term low-dose oral adefovir and already exhibited proteinuria, hypophosphatemia, and hypouricaemia prior to ZA initiation, suggesting preexisting renal tubular damage. After ZA initiation, he developed hypokalemia, hyperchloremia, metabolic acidosis, and glycosuria, indicating aggravation of renal tubular injury. Concomitant use of ZA with other nephrotoxic agents has been shown to further increase the risk of renal injury.^[11]^ Differential diagnoses were comprehensively excluded. No other nephrotoxic agents were identified in Patient #1. In Patient #2, preexisting adefovir exposure likely contributed to tubular injury; however, the rapid deterioration following ZA initiation and the marked improvement after its discontinuation implicated ZA as the primary causative factor. A hereditary form of FS was considered unlikely due to the late onset and absence of a relevant family history. Renal tubular function improved following ZA withdrawal in both patients; thus, a diagnosis of ZA-induced FS was strongly considered.

FS is a rare disorder that is often underrecognized in clinical practice and easily misdiagnosed as osteoporosis, rheumatoid arthritis, or osteochondrosis.^[12]^ FS has been reported to develop within 1 month of ZA treatment in some cases.^[13]^ Patient #1 developed hypouricaemia 4 months after ZA initiation, with a diagnosis of FS established 2 years later. Patient #2 exhibited signs of progressive renal tubular injury 3 months after starting ZA, and was diagnosed with FS 4 years later. These findings highlight the need for increased clinical awareness and early recognition of FS.

ZA is the most potent third-generation bisphosphonate, and exerts its effects by inhibiting osteoclast activity and inducing osteoclast apoptosis.^[2]^ ZA is excreted unchanged by the kidneys and exhibits a prolonged half-life in bone tissue. Its nephrotoxicity is both dose and time-dependent.^[14]^ The nephrotoxic effects of ZA may result from fluid-phase endocytosis-mediated internalization, induction of glutathione biosynthesis, and disruption of the tricarboxylic acid cycle. These processes can promote overproduction of reactive oxygen species, oxidative stress, and inflammation, ultimately resulting in renal injury.^[4,14]^ ZA-induced nephrotoxicity may also involve pharmacologic inhibition of the mevalonate pathway. Metabolomic and proteomic analyses have shown that ZA activates a cascade of dysregulated pathways, including TGFβ/Smad3-mediated profibrotic signaling, abnormal fatty acid metabolism, and impaired small GTPase signaling.^[4]^ This pathway-specific nephrotoxicity has been linked to upregulation of the fatty acid transporter SLC27A2 and impaired β-oxidation of fatty acids following ZA exposure. However, the precise mechanism by which ZA induces FS remains unclear.

The treatment of FS primarily targets the underlying cause, with adjunctive therapy addressing electrolyte disturbances and associated bone disease. Excessive phosphate loss impairs bone mineralization; therefore, patients require oral phosphate supplementation, a phosphate-rich diet, and administration of vitamin D and calcitriol. Renal tubular acidosis necessitates high-dose alkali therapy, and potassium supplementation is often required. Aminoaciduria, glycosuria, and proteinuria are typically asymptomatic and do not necessitate specific treatment. In this report, both patients exhibited varying degrees of renal tubular recovery following the discontinuation of ZA, consistent with findings reported in previous literature.^[4,11,13]^

## 4. Conclusion

Current literature provides no clear recommendations on the optimal timing for monitoring renal tubular function in patients undergoing ZA therapy. Based on our findings, we recommend that patients undergo renal function testing: including serum electrolyte levels, urinalysis, and blood gas analysis: within the first month after initiating ZA therapy. Continued close monitoring is essential thereafter. ZA should be promptly discontinued if renal tubular dysfunction is detected, as this may indicate the onset of secondary FS and should raise clinical suspicion among healthcare providers.

## Author contributions

**Conceptualization:** Yanrong Chen, Jinhui Ma, Yuqi Li, Yao Zhang.

**Methodology:** Yanrong Chen, Yao Zhang.

**Supervision:** Tao Ma, Yao Zhang.

**Writing – original draft:** Yanrong Chen, Jinhui Ma, Yuqi Li, Lili Qiao.

**Writing – review & editing:** Yanrong Chen, Jinhui Ma, Yao Zhang.
